# Noninvasive Temporal Interference Stimulation of the Subthalamic Nucleus in Parkinson's Disease Reduces Beta Activity

**DOI:** 10.1002/mds.30134

**Published:** 2025-04-09

**Authors:** Martin Lamoš, Martina Bočková, Florian Missey, Claudia Lubrano, Mariane de Araújo e Silva, Jan Trajlínek, Ondřej Studnička, Pavel Daniel, Romain Carron, Viktor Jirsa, Jan Chrastina, Radim Jančálek, Eric Daniel Glowacki, Antonino Cassara, Esra Neufeld, Irena Rektorová, Adam Williamson

**Affiliations:** ^1^ Brain and Mind Research Program, Central European Institute of Technology Masaryk University Brno Czechia; ^2^ First Department of Neurology Masaryk University School of Medicine, St. Anne's Hospital Brno Czechia; ^3^ Neuromodulation Technology Research, International Clinical Research Center St. Anne's University Hospital Brno Czechia; ^4^ Institut de Neurosciences des Systèmes Aix‐Marseille University and INSERM Marseille France; ^5^ Medico‐Surgical Unit Epileptology, Functional and Stereotactic Neurosurgery Timone University Hospital Marseille France; ^6^ Department of Neurosurgery Masaryk University School of Medicine, St. Anne's Hospital Brno Czechia; ^7^ Bioelectronics Materials and Devices, Central European Institute of Technology, Brno University of Technology Brno Czechia; ^8^ Foundation for Research on Information Technologies in Society Zurich Switzerland

**Keywords:** temporal interference stimulation, deep brain stimulation, subthalamic nucleus, Parkinson's disease, beta power, local field potentials

## Abstract

**Background:**

Temporal interference stimulation (TIS) is a novel noninvasive electrical stimulation technique to focally modulate deep brain regions; a minimum of two high‐frequency signals (*f*_1_ and *f*_2_ > 1 kHz) interfere to create an envelope‐modulated signal at a deep brain target with the frequency of modulation equal to the difference frequency: Δ*f* = |*f*_2_ – *f*_1_|.

**Objective:**

The goals of this study were to verify the capability of TIS to modulate the subthalamic nucleus (STN) with Δ*f* and to compare the effect of TIS and conventional deep brain stimulation (DBS) on the STN beta oscillations in patients with Parkinson's disease (PD).

**Methods:**

DBS leads remained externalized after implantation, allowing local field potentials (LFPs) recordings in eight patients with PD. TIS was performed initially by two pairs (*f*_1_ = 9.00 kHz; *f*_2_ = 9.13 kHz, 4 mA peak‐peak per pair maximum) of scalp electrodes placed in temporoparietal regions to focus the envelope signal maximum (Δ*f* = 130 Hz) at the motor part of the STN target.

**Results:**

The comparison between the baseline LFPs and recordings after TIS and conventional DBS sessions showed substantial suppression of high beta power peak after both types of stimulation in all patients.

**Conclusions:**

TIS has the potential to effectively modulate the STN and reduce the beta oscillatory activity in a completely noninvasive manner, as is traditionally possible only with intracranial DBS. Future studies should confirm the clinical effectiveness of TIS and determine whether TIS could be used to identify optimal DBS candidates and individualize DBS targets. © 2025 The Author(s). *Movement Disorders* published by Wiley Periodicals LLC on behalf of International Parkinson and Movement Disorder Society.

Temporal interference stimulation (TIS) is a novel noninvasive electrical brain stimulation technique that has the potential to modulate deep brain regions without modulating shallower cortical regions.^1^, ^2^ The focal modulation of TIS is possible through the use of multiple high‐frequency sources (*f* > 1 kHz) applied noninvasively with the standard scalp electrodes used for transcranial stimulation. The technique exploits the observation that neuronal membranes have a higher stimulation threshold for kilohertz frequencies limiting depolarization and stimulation properties above these frequencies as compared with low frequencies and amplitude‐modulated signals.^3^, ^4^, ^5^ For TIS, a minimum of two high‐frequency electric fields (*f*_1_ and *f*_2_) are used, where the difference in frequency (Δ*f* = |*f*_2_ − *f*_1_|) is the neuromodulation frequency at the deep brain target. Although TIS is a relatively new noninvasive method, several applications for brain stimulation in humans have been successfully demonstrated. TIS focally modulated hippocampal activity and enhanced the accuracy of episodic memory in healthy subjects.^6^ In another study, striatal TIS showed the enhancement of motor performance in healthy older participants.^7^

Deep brain stimulation (DBS) is a well‐established clinical neuromodulation therapeutic approach based on surgical electrode implantation to specific brain targets and the delivery of constant or intermittent electrical stimulation from an implanted electrode.^8^ The method has been successfully used to treat various neurological and psychiatric disorders; DBS therapy modulates pathological neural activity and circuits.^9^ Specifically, in Parkinson's disease (PD), which is the most frequent indication for DBS surgery, the main and most well‐characterized oscillatory activity is the beta power hypersynchrony (13–35 Hz) in the motor circuits, which correlates to the severity of motor symptoms, ie, hypokinesia and rigidity.^10^ This activity is suppressible by dopaminergic treatment and by DBS.^11^, ^12^, ^13^, ^14^ The subthalamic nucleus (STN) is the most common DBS therapeutic target used in the majority of patients with PD.

The aim of this study was to evaluate the potential of TIS to effectively modulate the STN activity and to compare the neuromodulatory performance of TIS with conventional DBS on PD‐related beta oscillations in patients.

## Subjects and Methods

### Subjects

Patients with PD with externalized DBS leads implanted in the STN bilaterally (n = 8; see Table 1) in the immediate postoperative period (second day after surgery) before the system internalization (connection to the implantable pulse generator) participated in the study. All subjects were informed about the scientific nature of this study and signed informed consent forms. The study received the approval (IIT/2023/25) of the local ethics committee (the ethics board of St. Anne's hospital in Brno). During the procedure, patients were in the medication *off* state, after 12 hours of dopaminergic therapy withdrawal. Subjects reclined comfortably in the monitoring bed, in a Faraday‐shielded room. They were instructed to remain calm and to avoid unnecessary movements.

**TABLE 1:** Patient characteristics

ID No.	Sex	Age (y)	Disease duration (y)	LED (mg)	Symptom severity dominant side	DBS system (IPG, leads)
1	M	64	14	1601	Right	Percept, B33005
2	M	53	10	1604	Left	Infinity, 6172
3	F	75	10	1291	Right	Percept, B33005
4	F	67	14	1368	Right	Percept, B33005
5	M	60	8	1480	Right	Percept, B33005
6	M	55	9	1810	Left	Percept, B33005
7	F	58	6	1615	Left	Percept, B33005
8	F	55	12	1091	Right	Percept, B33005

Abbreviations: LED, levodopa‐equivalent dose; DBS, deep brain stimulation; IPG, implantable pulse generator.

### Surgical Procedure and Leads Localization

The DBS leads were implanted bilaterally into the STN by a frame‐based stereotactic magnetic resonance imaging (MRI)‐guided technique, including intraoperative microelectrode recording and stimulation. After the implantation of both leads, a computed tomography (CT) scan was performed under stereotactic conditions covering the entire length of the implanted leads. Contact positions were localized using Lead‐DBS software^15^ (https://www.lead-dbs.org/). Preoperative MRIs were coregistered to postoperative CT using rigid transforms from Advanced Normalization Tools (ANTs).^16^ The normalization to the Montreal Neurological Institute (MNI) space by ANTs was complemented by a brain shift correction to compensate for brain movement caused by pneumocephalus. Each step was manually checked in each patient. The information about the lead position served in selecting the stimulating contacts under the conventional DBS conditions and for knowledge about the precise locations where local field potentials (LFPs) were registered.

### Experimental Protocol and Recordings

LFPs were recorded from the STN in the medication *off* state via externalized DBS leads during

1. baseline 5 minutes of resting state,
2. TIS for 3 minutes (and the following 3 minutes of resting state),
3. conventional therapeutic DBS for 3 minutes (and the following 3 minutes of resting state),
4. sham 3‐minute sessions with bilateral 9‐kHz stimulation with/without ramp up and down (in subject 7), and
5. TIS for 3 minutes focused out of the STN to the area of the occipital lobe and nontherapeutic low‐frequency (30 Hz) STN TIS for 3 minutes (in subject 8).

All experimental sessions were separated by a 10‐minute pause during which LFPs were also registered; for technical reasons due to the high sampling rate, they were not registered for the whole time period. Sham sessions were measured between baseline and stimulation conditions. In subjects 7 and 8, the partial MDS‐UPDRS III (International Parkinson and Movement Disorders Society–Unified Parkinson's Disease Rating Scale Part III) scores were administered to evaluate the patient's clinical condition and the severity of motor symptoms during the different stimulation conditions.

The data were recorded by amplifier M&I BioSDA09 (M&I Ltd, Prague, Czech Republic) with sampling frequency of 25 kHz/24 bit. Externalized leads and 10–20 scalp electrodes were recorded against the scalp Cz reference electrode. Intracerebral contacts used for the stimulation were temporarily disconnected from the amplifier to avoid input saturation when conventional DBS was ON.

### Electrical Stimulation

The stimulation was targeted at the STN contralateral to the dominant side of symptom severity in each patient.

**FIG. 1:**
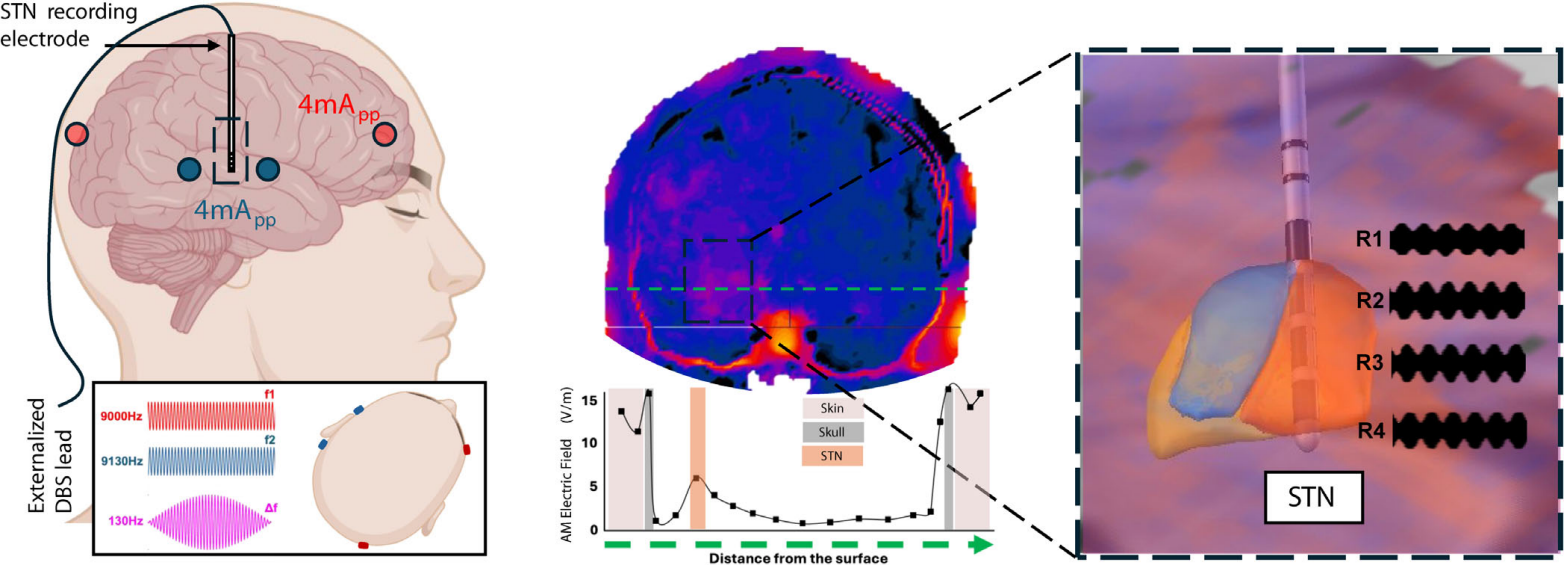
The principle of the temporal interference stimulation (TIS) targeting subthalamic nucleus (STN). (Left panel) Deep brain stimulation (DBS) electrodes are implanted in the STN, using a standard externalized process—the wires from the electrodes are connected to the battery in a subsequent surgery (leaving externalized wires for a 1‐day period, allowing access to each electrode individually). During the work, a recording is made from the DBS electrodes while stimulation is applied in three forms: DBS stimulation directly, TIS stimulation from transcutaneous electrodes, and sham stimulation (only high‐frequency carriers with no amplitude‐modulated signal from transcutaneous electrodes). (Center panel) An example of patient‐specific stimulation of the transcutaneous‐applied TIS, with a clear amplitude‐modulated signal maximum at the STN target. (Right panel) A zoom view of the targeted region showing the electrode placement with respect to the patient's STN. [Color figure can be viewed at wileyonlinelibrary.com]

TIS was performed by two pairs (*f*_1_ = 9.00 kHz; *f*_2_ = 9.13 kHz, 4 mA peak‐peak per pair maximum) of scalp silver chloride–plated carbon‐fiber electrodes placed in the frontoparietal regions to focus the maximum of 130 Hz interference envelope in the target STN closest to the electrophysiological sweet spot of good patient outcome (Fig. 1).^17^ Positions were modeled for each patient individually in the Sim4Life software package (ZMT, Zurich, Switzerland). The TIS was provided using a two‐channel Keysight EDU33212A generator connected to two Digitimer DS5 electrical stimulators.

The sham stimulation was carried out using the same scalp electrodes as used for TIS. The stimulation frequency was 9 kHz for both TIS channels (Δ*f* = 0) with an amplitude of 4 mA peak‐peak per channel and 3‐minute stimulation duration. Two variants of sham were performed in subject 7: with and without ramping. The sham variant with ramping included 1 minute of linear ramp‐up at the beginning and 1 minute of linear ramp‐down at the end of the 3‐minute stimulation session. Beta activity was not suppressed with sham, using the high‐frequency carriers alone. Both variations are shown in Fig. 3; however, we prefer to perform sham with a ramp to avoid any possible transient effects of neural membranes because of the step‐function nature of the instantaneous ON and OFF of the sham stimulation.^5^

To avoid any kind of possible risks of electrical field enhancement near the implanted DBS leads during TIS based on published simulations,^18^ we additionally performed the experimental measurements with a phantom (see Supporting Information Data S1). No focal charge enhancement by an implanted lead was found.

Conventional DBS was performed by a Medtronic or Abbott external DBS neurostimulator. Contacts closest to the sweet spot (in the motor part of the STN located dorsally) were selected for the bipolar stimulation with the following parameters: amplitude of 2 V, 90‐μs pulse width, and 130‐Hz frequency.

### Finite Element Modeling

To estimate TIS (Supporting Information Fig. S1), we performed finite element simulations using the Sim4Life and the TI Planning Tool, both developed by Zurich MedTech AG. These simulations incorporated an electro‐ohmic quasistatic solver to effectively solve the equation ∇σ∇ϕ = 0, where σ represents the local electrical conductivity, ω is the angular frequency, and the E‐field is calculated as E = −∇ϕ. By approximating Maxwell's equations, the dominance of ohmic currents over displacement currents was addressed, given that σ ≫ ωϵ and σ ≠ 0, thus confining E‐field calculations to the lossy domain.

The human model used in Sim4Life simulations was constructed from patient‐specific MRI (T1) and coregistered CT scans. Each patient's head model included the implanted DBS electrodes. Tissue and electrode properties, such as conductivity and permittivity, were automatically assigned based on the IT'IS Foundation database.^19^ Stimulation electrodes were designed to replicate the dimensions of gel‐based electrodes used in human experiments. Data, normalized to the total current, were obtained from stimulation sessions at active electrodes under Dirichlet boundary conditions. The maximum envelope modulation amplitude was calculated using equations from the study by Grossman and colleagues.^2^

### Data Analysis

The LFP data were processed offline using MATLAB 2021a (The MathWorks, Natick, MA, USA) and the FieldTrip toolbox.^20^ Signals from macrocontacts on externalized leads were recalculated to a bipolar montage to exclude the volume conduction from other structures and to confirm the local origin of the potentials.^21^, ^22^ The bipolar montage created three bipolar signals for the left STN (L0‐L1, L1‐L2, and L2‐L3) and similarly for the right STN (R0‐R1, R1‐R2, and R2‐R3). Bad signals and segments were detected and marked manually by visual inspection of the data in SignalPlant software^23^ and excluded from the subsequent analysis. Bipolar signals from the STN contralateral to the dominant side of symptom severity and closest to the sweet spot were analyzed using the frequency‐dependent evaluation. Power spectra were analyzed in randomly selected 60‐second segments of the baseline condition and immediately in the first 60 seconds of the resting state after the 3‐minute stimulation (TIS or DBS) sessions, to avoid the influence of the stimulation artifacts on the LFPs.

LFPs were filtered from 1 to 100 Hz using a second‐order Butterworth filter in forward and reverse directions for zero‐phase distortion. The ongoing signals were then segmented into 2‐second parts. The power spectrum was analyzed by discrete Fourier transforms complemented by Hanning multitapering with 2‐Hz smoothing. The spectral resolution was 0.25 Hz. Finally, the oscillatory part of the power spectrum was extracted as a difference between the original frequency spectrum and its aperiodic component computed by the FOOOF (fitting oscillations and one‐over‐F) approach.^24^ The time evolution of the oscillatory beta power after the stimulation condition was computed by the same pipeline using a sliding window approach with 10‐second steps. Beta oscillatory power was evaluated as a mean power of the oscillatory part of spectrum in an interval ±2 Hz around the maximum of the patient‐specific beta peak.

The resting‐state oscillatory powers of LFPs under different conditions were compared with the main focus on the pathological beta power peak changes using the Wilcoxon signed‐rank paired test.

The amplitude of the interference envelope during the TIS in both STNs was computed as the difference between the minimal and maximal amplitudes in the absolute value of the LFP signal's Hilbert transform.

## Results

**FIG. 2:**
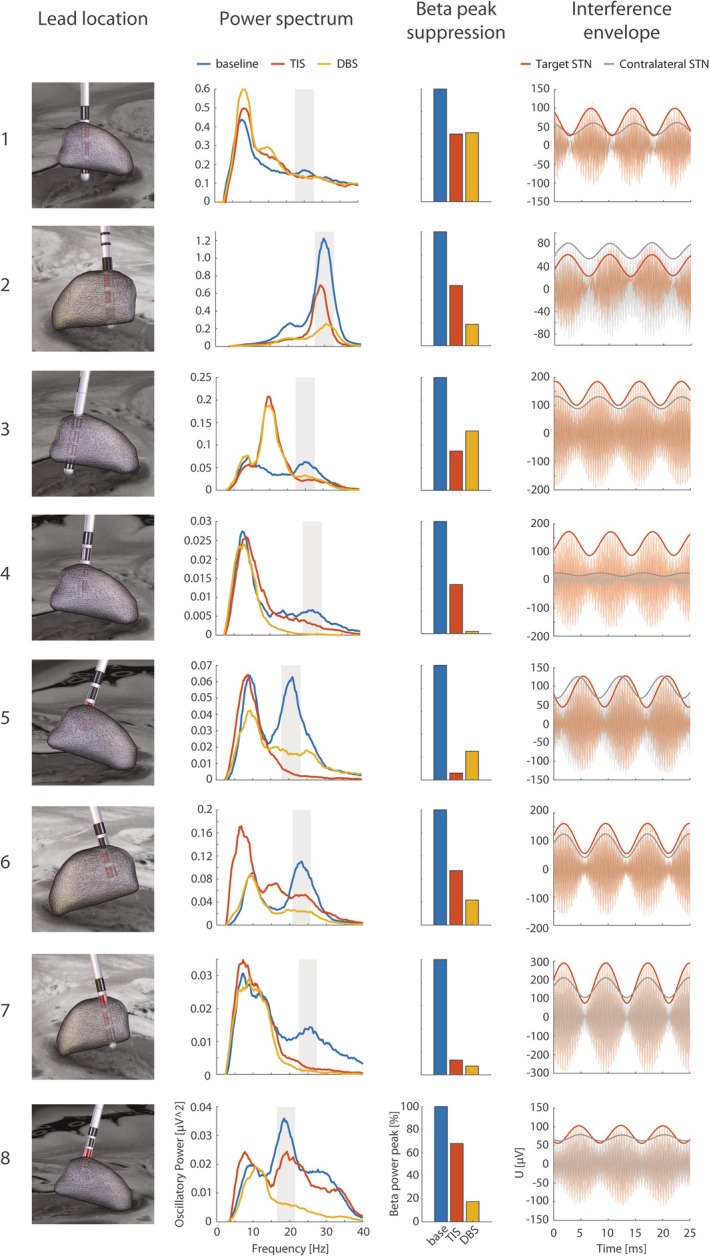
The effect of stimulation on subthalamic nucleus (STN) beta oscillations in eight subjects. The first column shows the lead location in the analyzed STN. Contacts closest to the STN sweet spot selected for deep brain stimulation (DBS) and LFP sensing are marked in red. The second column presents the oscillatory part of the power spectrum under the baseline, temporal interference stimulation (TIS), and DBS conditions. The area of interest, where the pathological beta peak can be seen, is marked in gray. The third column expresses the reduction of the pathological beta peak power under the TIS and DBS conditions as compared with the baseline. The fourth column shows the interference envelope in the target and contralateral STN. [Color figure can be viewed at wileyonlinelibrary.com]

The optimal contacts for stimulation and LFP sensing were chosen based on the symptom severity dominant side and based on the distance to the STN sweet spot as defined by Horn and colleagues.^17^ Thus, in each subject, the nucleus contralateral to the symptom severity dominant side was selected as a target STN, and contacts closest to the MNI coordinates, *x* = ±14.4, *y* = −13.2, *z* = −4.9 mm, were used for conventional DBS stimulation and LFP recording (Fig. 2). The difference in the distance between the first and second closest contact to the sweet spot was significant across patients (Wilcoxon signed‐rank test, *P* = 0.012, *z* = 2.521, effect size *r* = 0.891).

Conventional DBS led to a significant beta power reduction in the STN compared with the baseline condition, as expected, in all subjects (Wilcoxon signed‐rank test, *P* = 0.012, *z* = 2.521, effect size *r* = 0.891) (Fig. 2). A similar trend was observed for TIS compared with the baseline, and the beta power decrease was significant (Wilcoxon signed‐rank test, *P* = 0.012, *z* = 2.521, effect size *r* = 0.891) (Fig. 2). There was no significant difference between TIS and DBS conditions (Wilcoxon signed‐rank test, *P* = 0.263, *z* = 1.120, effect size *r* = 0.396). The amplitude and frequency of the pathological beta power peak differed among patients but were presented mainly in higher beta ranges around 25 Hz (Fig. 2). The higher beta power reduction after DBS and TIS was followed in some subjects (subjects 1, 3, and 6) by a power increase in the lower frequencies, usually in alpha/low beta borderline (Fig. 2).

Interfering stimulation signals in both STNs could be compared in high‐sampled LFP recordings of the TIS condition (Fig. 2). The significantly higher amplitude of the interference envelope in the target STN as compared with the contralateral side (Wilcoxon signed‐rank test, *P* = 0.012, *z* = 2.521, effect size *r* = 0.891) showed the possibility of effectively steering the stimulation to the region of interest.

DBS and TIS were both well tolerated in all subjects. No adverse effects were observed, and only some mild side effects occurred, mainly common transient limb paresthesias during DBS and local paresthesias on the head surface during TIS (in two subjects: subjects 1 and 2). Despite targeting the motor part of the STN, we did not induce choreatic dyskinesias, because the stimulation sessions were brief and the subjects were in the *off* medication condition.

**FIG. 3:**
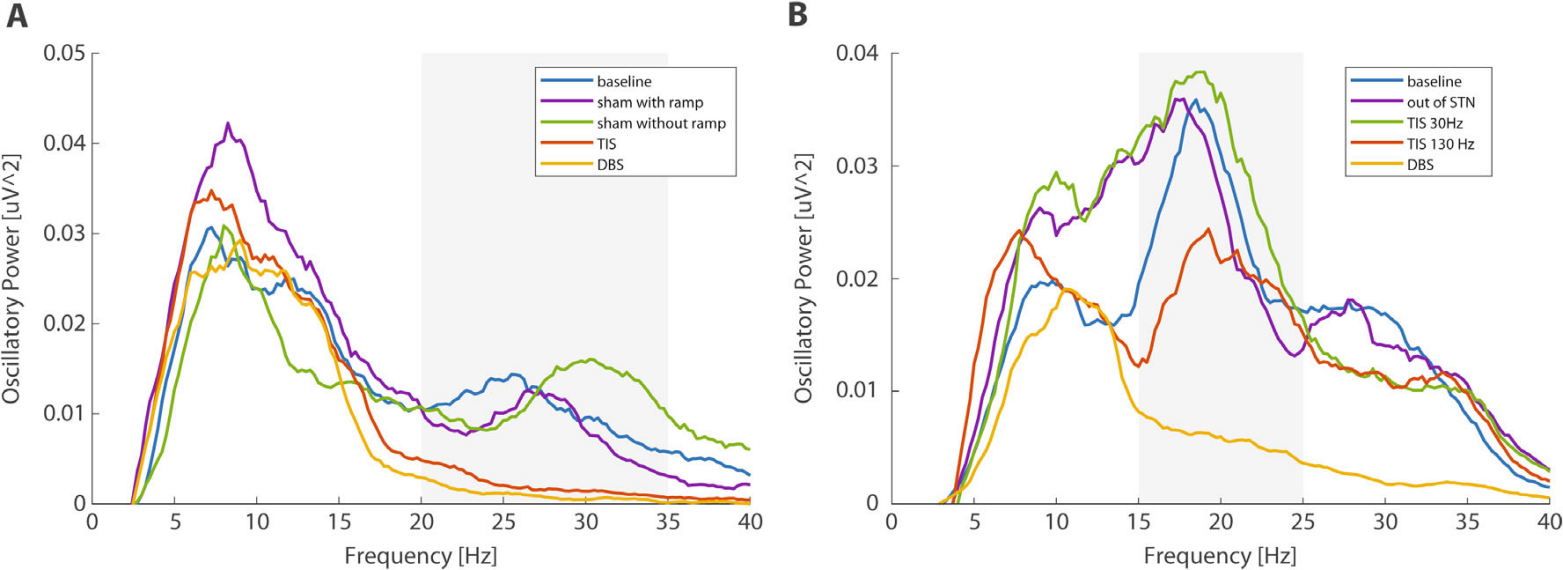
The effect of control conditions on subthalamic nucleus (STN) beta oscillations. Case example of subject no. 7's (**A**) oscillatory part of the power spectrum under the baseline, deep brain stimulation (DBS), temporal interference stimulation (TIS), and both sham conditions. Sham stimulation, the utilization of carrier frequencies alone (no Δ*f*), does not reduce the beta power. Application of sham without a ramp (instantaneous on) seems to increase beta. A proper ramp ensures no transient artifacts. Case example of subject 8's (**B**) oscillatory part of the power spectrum under baseline, DBS, STN TIS 30 Hz, STN TIS 130 Hz, and occipital lobe TIS 130 Hz. Targeting another structure out of STN does not modulate beta peak in STN. A similar effect can be seen also for the 30‐Hz STN TIS condition. [Color figure can be viewed at wileyonlinelibrary.com]

There was an opportunity to perform sham stimulations, of target stimulation, and nontherapeutic frequency TIS sessions in subjects 7 and 8. In subject 7, we focused on sham stimulations that contained only high‐frequency stimulation (Δ*f* = 0) with and without ramping. Compared with the DBS and TIS conditions, no effects on beta power were observed for the sham condition (Fig. 3A). Partial MDS‐UPDRS III scores (without gait and balance evaluation) were baseline MDS‐UPDRS score of 30 points with no influence from the sham stimulation, MDS‐UPDRS score after TIS was 26 points, and MDS‐UPDRS score after DBS was 18 points.

In subject 8, we focused on the possible influence of the therapeutic 130‐Hz TIS out of the area of the STN (targeted to the left occipital lobe; Supporting Information Fig. S2) and the nontherapeutic low‐frequency (30‐Hz) STN TIS. Neither of these sessions led to STN beta power suppression (Fig. 3B). The baseline partial MDS‐UPDRS III score was 28 points, with no influence by TIS of the STN and 30‐Hz STN TIS; the score after 130‐Hz STN TIS was 26 points, and it was 20 points after conventional DBS.

**FIG. 4:**
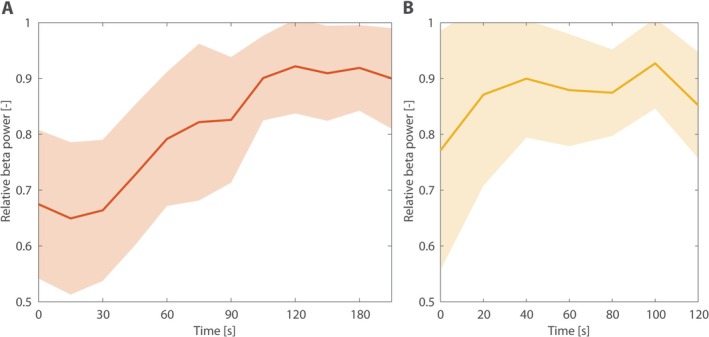
Time evolution of beta power after the temporal interference stimulation (TIS) (**A**) and deep brain stimulation (DBS) (**B**) conditions. Beta power was evaluated as a mean power of the oscillatory part of the spectrum in the interval ±2 Hz around the maximum of patient‐specific beta peak normalized across patients. Solid line expresses the mean, and background color corresponds to the standard deviation across patients. [Color figure can be viewed at wileyonlinelibrary.com]

Time‐frequency analysis of the oscillatory part of the beta power peak was calculated in recordings registered after the TIS and DBS conditions to follow the time evolution of the beta suppression (approximately 3 minutes for TIS and 2 minutes for DBS). The TIS aftereffect lasted approximately 2 minutes (Fig. 4A). For the DBS condition, the reactivity varied across the group and in some patients was more immediate than TIS (Fig. 4B).

## Discussion

Electrical stimulation using TIS with two different high‐frequency (kHz) sinusoidal electric fields enabled noninvasive DBS of the STN in patients with PD scheduled for standard deep brain implantation. TIS created an amplitude modulation signal with an envelope equal to 130 Hz, in the neural response range for standard stimulation, at the target brain region.^2^, ^25^, ^26^, ^27^ TIS has been demonstrated as functionally influencing neurons in deep brain structures such as in the hippocampus in animal models without recruiting neurons of the overlying cortex.^2^, ^28^, ^29^ The ability to evoke a clinical response such as seizure‐like events similar to the stimulation via implanted intracranial stereoelectroencephalography (SEEG) electrodes has been presented in epilepsy surgery candidates to explore the epileptogenic zone.^30^ An interesting result from the perspective of PD is that TIS can potentially influence the neurochemical processes in the brain leading to an evoked phasic dopamine release in the rat striatum,^31^ because the dopaminergic deficit plays a major role in the PD main motor symptoms development.

The same TIS technique was used in two recent studies with healthy human subjects; clinically effective hippocampal noninvasive DBS led to improved accuracy of episodic memory^6^ and increased activity in the striatum associated with enhanced motor performance in healthy older participants.^7^ The potential effect of TIS on local field oscillatory activity in the gamma frequency band in deep brain regions has been described on animal hippocampal slices and validated in a model of the human head.^32^ Moreover, TIS has been proposed, based on a primate study, as an ideal tool for disrupting pathological oscillatory activity, a hallmark of many neurological disorders.^33^

In the field of PD, a very recent study with human subjects presented the effect of TIS targeting the globus pallidus internus (GPi) on motor symptoms evaluated by the MDS‐UPDRS III scale.^34^ This pilot study suggested that TIS is capable of alleviating motor symptoms, mainly bradykinesia and tremor.

The novel findings presented in this article confirm the ability of TIS to reach a small‐sized deep brain region like the STN in a group of patients with PD. To the best of our knowledge, this is the first study to show direct evidence of the TIS effect on oscillatory activity related to PD motor symptoms in humans. This exciting new method influences the disease‐specific pathological LFPs toward more physiological patterns in a similar way as DBS, which is routinely used for symptomatic treatment in various neurological and psychiatric disorders.

Oscillations in the beta frequencies are crucial in motor control in corticobasal ganglia loops^35^ and have physiological and pathological roles. Beta power is known to be suppressed before self‐paced and cued voluntary movements.^36^, ^37^ Increased and hypersynchronized beta oscillations have been found via intracranial recordings to correlate to the main PD signs: bradykinesia and rigidity.^11^, ^12^, ^13^ Oscillations in the beta frequencies serve as a well‐known and the most established DBS marker in patients with PD.^35^ Successful clinical trials using adaptive DBS based on beta power sensing^38^, ^39^ further confirm that beta power modulation underpins the clinical effects of DBS.

In our study, the power reactivity was observed in all our subjects in high beta (between 20 and 30 Hz) ranges; these ranges were recently described in detail as linked solely with the DBS effect.^40^ Because dopaminergic treatment reduces mainly low beta power, the sometimes observed alpha/low beta power increase after DBS/TIS in our patients was very probably caused by the dopaminergic medication withdrawal during the measurements. Therefore, visible TIS modulation corresponds to the well‐known effect of DBS, which was also replicable in this study. To exclude the lasting influence of DBS on the STN LFPs activity during TIS, TIS sessions always preceded the DBS in our experimental protocol.

TIS is a different type of neuromodulation, applied in a sinusoidal pattern at a subthreshold intensity; DBS is a pulsed pattern suprathreshold intensity stimulation that generates action potentials.^41^ Despite these different mechanisms of action, there is growing evidence that TIS has the potential to influence deep brain oscillatory activity and induce clinical effects in a way similar to DBS.^29^, ^34^ It is possible to speculate that the unifying ground of modulatory effects might be caused by employing higher stimulation frequencies, promoting prokinetic gamma rhythms in motor circuits.^42^

Similar to the other previous human TIS studies, the stimulation was well tolerated and did not lead to any serious adverse effects and did not unintentionally affect any other brain regions. The sometimes‐observed minor side effects were comparable with the clinical DBS side effects and disappeared immediately after the stimulation was turned *off*. The favorable tolerability and safety profile of TIS targeting the striatum or hippocampus, including young and older adults and patients with traumatic brain injury, was shown in a recent study on a large dataset.^43^

The main limitation of this study was our inability to correlate the stimulation outputs and beta power changes to the clinical state, because we could not administer the full MDS‐UPDRS scale. Patients were on the first day postsurgery, not verticalized, and transported from the postoperative intensive care unit to the research area. To objectively validate the motor symptom improvement, we planned to measure the tremor intensity using accelerometers, but probably because of the well‐known microlesional effect, the tremor in our group of patients was only minor and not constant, and the other motor symptoms (bradykinesia and rigidity) were also reduced.

Although our study was not designed to evaluate the clinical aftereffects of TIS, we made efforts to evaluate at least partial clinical effects in two patients. The two case‐based observations in subjects 7 and 8 showed an immediate mild improvement in partial MDS‐UPDRS III after TIS, which was less prominent than that of DBS. Notably, we observed no influence of sham stimulations, out‐of‐target TIS, or low‐frequency nontherapeutic TIS on either LFPs or the patient's clinical condition as assessed by partial MDS‐UPDRS III. However, further studies with this experimental design are necessary.

It is well‐known from clinical practice that the duration of DBS stimulation to improve the clinical motor symptoms strongly varies in different diagnoses, from immediate effects in essential tremor to weeks or months in dystonias.^44^ In patients with PD, an immediate effect of DBS is not usually seen; the effect occurs approximately in terms of hours and is highly variable among individual patients. Subthreshold TIS compared with suprathreshold DBS^41^ would probably require even longer stimulation to reach clinical effectiveness. Because our stimulation sessions lasted only 3 minutes, we were not able to document convincing clinical improvement of the PD motor symptoms in contrast with a recent study,^34^ in which a 20‐minute TIS session targeting the right GPi was used in patients with PD. A recent study on an in vitro “TIS on a chip” setup using rat cortical neurons on microelectrode arrays demonstrated that TIS elicited neuronal electrophysiological responses mainly 24 hours after stimulation.^45^

Another limitation is the number of subjects involved in the study; the collection of this valuable but rare data is challenging. Even though the effect size is large, the results of the statistical tests must be interpreted carefully. Future studies must focus on larger cohorts, longer TIS delivered at multiple sessions, and clinical‐state correlations.

To conclude, DBS in general is a very successful therapy in various movement disorders indications. However, the individual clinical outcome varies among patients, and the future benefit is always difficult to predict. Choosing the best therapeutic target is also sometimes challenging, mainly in essential tremor or tremor‐dominant PD, where the STN and ventral intermediate nucleus of the thalamus are both possible therapeutical targets. Because the stimulation effects on tremor symptoms are usually immediate, this could be an example, in which TIS could greatly improve clinical practice and refine DBS indication criteria, because the different targets and the future clinical effects of the stimulation could be tested in advance.

We showed that TIS of the motor part of the STN suppresses beta oscillations in a similar way to that of DBS, which may have major clinical implications. Future studies have to assess whether TIS could be used in selecting optimal DBS candidates and brain targets, and whether it has the potential to identify new DBS targets and approaches in a totally noninvasive manner.

## Author Roles

Conceptualization: M.L., M.B., I.R., and A.W.; methodology: M.L., C.L., F.M., and A.W.; finite element simulations: F.M.; simulations—out of STN target: A.C. and E.N.; formal analysis: M.L.; field enhancement experiments: E.D.G.; investigation: M.B., M.L., C.L., M.d.A.e.S., J.T., and O.S.; surgical procedures: J.C. and R.J.; resources: M.B., I.R., and A.W.; data curation: P.D.; writing—original draft: M.B. and M.L.; writing—review and editing: all authors.

## Full financial disclosures of all authors for the preceeding 12 months

A.C. and E.N. are shareholders of TI Solutions AG, a company dedicated to producing temporal interference (TI) stimulation devices to support TI research.

## Supporting information


**Data S1.** Supporting Information.

## Data Availability

The data that support the findings of this study are available on request from the corresponding author. The data are not publicly available due to privacy or ethical restrictions.
