# Supplementary material for: Noninvasive Temporal Interference Stimulation of the Subthalamic Nucleus in Parkinson's Disease Reduces Beta Activity
Source: Mov Disord. 2025 Apr 9;40(6):1051–60. doi: 10.1002/mds.30134 (PMC12160966; doi:10.1002/mds.30134)
Supplement: Supplementary file 1 — Data S1. Supporting Information. [file MDS-40-1051-s001.docx]

**Non-invasive temporal interference stimulation of the subthalamic nucleus in Parkinson’s disease reduces beta activity**

Supplementary material

Electrical field modelling


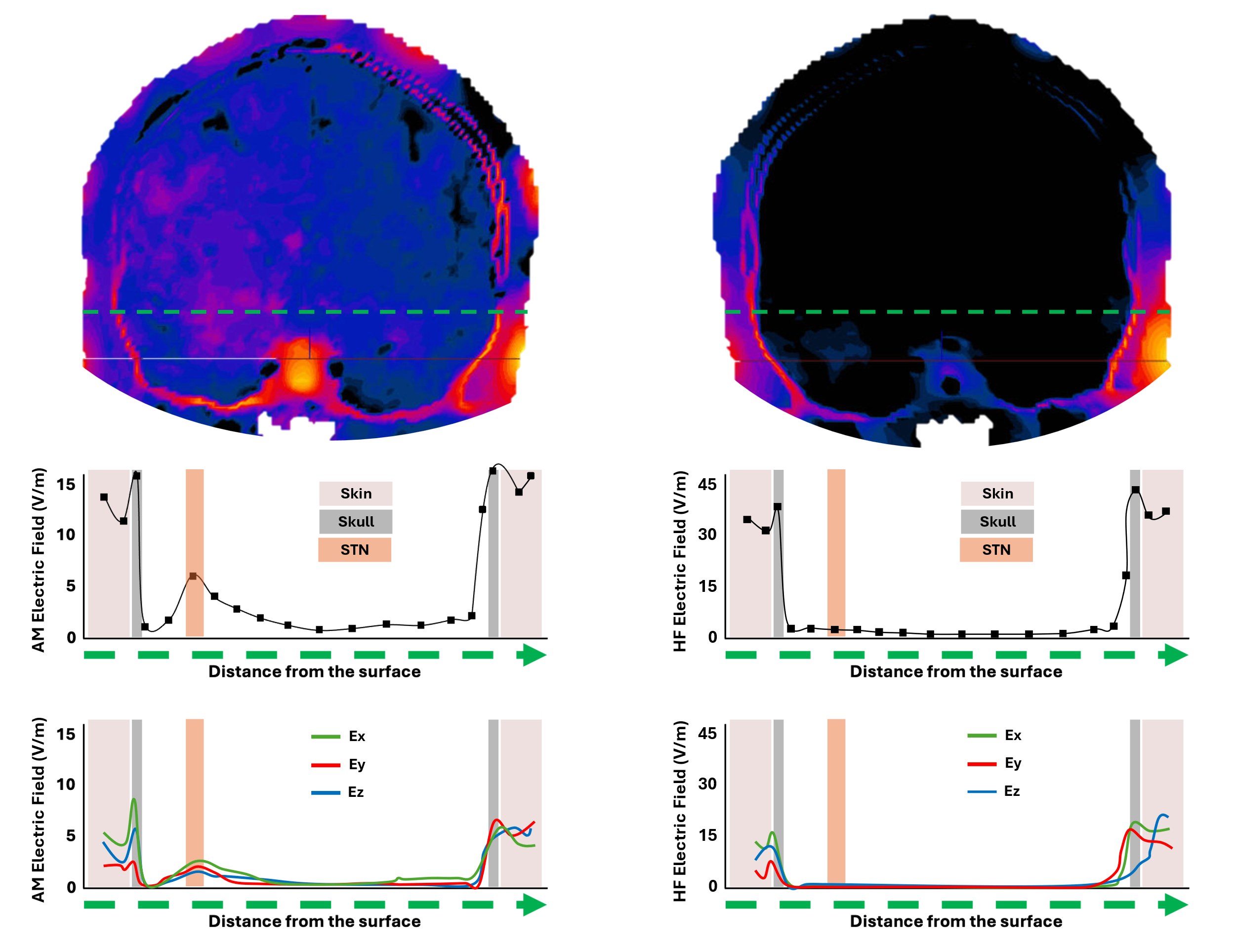


*Figure S1: Simulation of the distribution of AM and HF electric fields in the human brain for STN-targeted TI stimulation.*


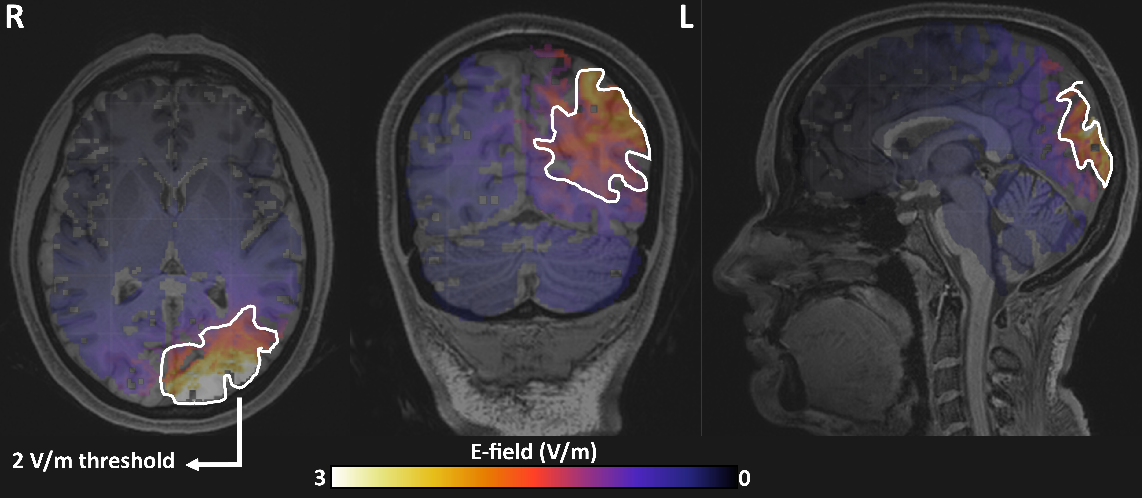


*Figure S2: Patient-specific simulation of the E-field for the TIS session focused out of the STN (occipital lobe).*

Field enhancement testing

The presence of implanted metal electrodes invites the hypothesis that the presence of conductors may significantly affect the electrical potential profile of noninvasively-applied stimulating E-fields. We believe that the magnitude of such effects is very unlikely to influence the outcome of stimulation, at least when considering the electric fields involved. It is important to note that during the application of electrical stimulation, the implanted electrodes are connected externally to a high-impedance recording amplifier, and thus can be considered electrically floating. The possibility of the capacitive coupling of electric currents that would pass via the implanted electrodes to a return electrode and stimulate the intervening tissue can therefore be discounted.

The presence of floating conductors can affect the E-field nonetheless, as was recently suggested by Cassara et al. and Karimi et al. To understand the possible magnitude of this effect on our experiment, we set up a simple phantom experiment (Figure S2), in which we applied stimulation currents to a dilute buffer saline solution and recorded the electric field using a bipolar probe (interprobe distance = 1 mm). We then used an *xyz* micromanipulator stage to vary the position of the bipolar probe with respect to a DBS lead which was placed into the bath. We applied a sufficient stimulation current (0.15 mA in our model) to reach E-fields in the range of suprathreshold stimulation (20 V/m) in the center of the electrolyte bath.


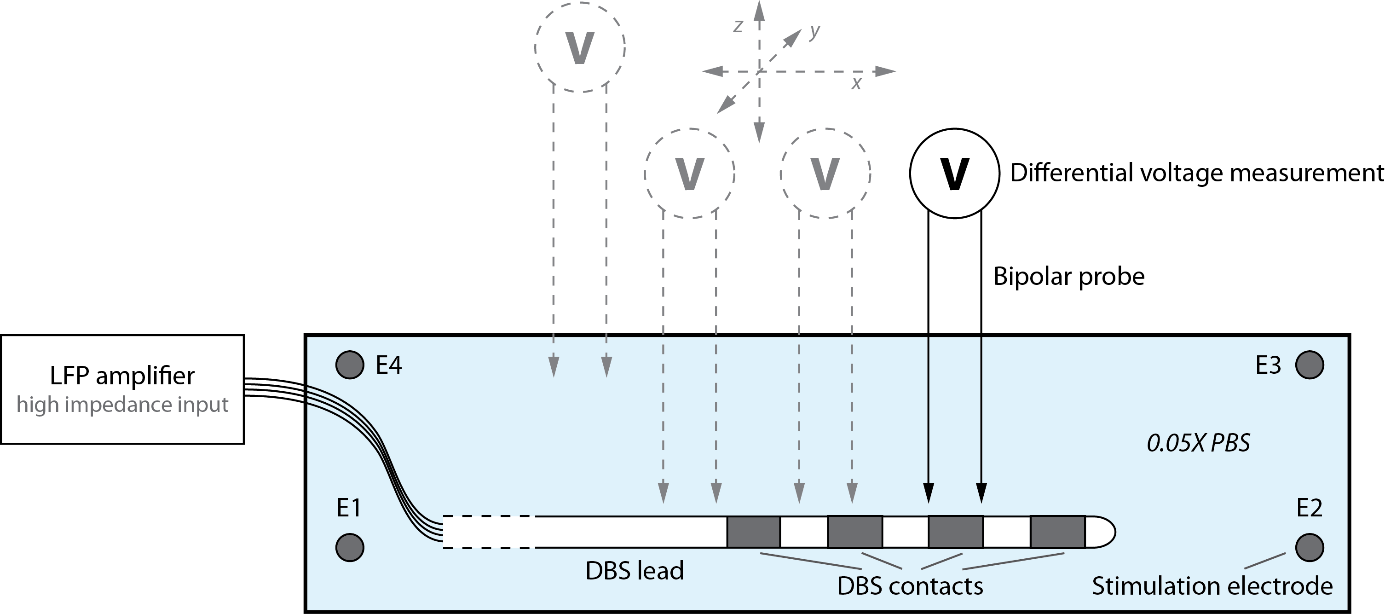


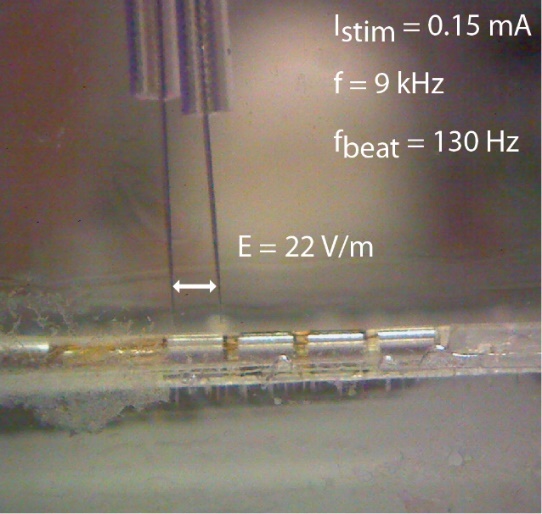


*Figure S2: Recording of a local E-field in relation to a DBS lead in a phantom model. The E-field was quantified using a bipolar probe consisting of a pair of insulated tungsten microwires with a 20-micron exposed tip (World Precision Instruments, catalog number WE3003X.XA3). Stimulation currents were applied using Ag/AgCl wires in a phosphate buffered saline diluted down to 0.05X. The DBS lead was fixed in place inside of the batch and the relative positions of the recording probe electrodes were varied using a Thorlabs nanomaster xyz stage, with the position monitored using a USB microscope. Voltage recordings were obtained using PicoScope 4262 in the differential mode, in which the recording probe electrodes were connected to channels A and B and A-B was recorded to reject common noise and provide an accurate bipolar E-field value.*

Under no circumstances did we observe any E-field enhancement effects near the DBS lead electrode surface (Table S1). In fact, placing the bipolar electrodes within a few hundred micrometers away from the DBS electrodes predictably caused an attenuation of the local E-field by roughly 10-15%, since the DBS electrode surface is more conductive than the surrounding electrolyte medium. Varying the position of respective stimulation electrode pairs and the DBS/bipolar recording electrodes never produced any kind of enhancement effect.

*Table S1: E-field intensity under various conditions of probe position and stimulating E-field orientation. “Above contact” refers to the probe position directly above one of the DBS lead contacts. “Above lead” refers to the probe position above the insulated part of the DBS lead. Orientation 0° indicates that stimulating electrodes (E1-E2) are in line with the DBS lead trajectory. Orientation 45° indicates that stimulating electrodes (E1-E3) are under 45° to the DBS lead trajectory.*

|  |  | E-field intensity [V/m] | | |
| --- | --- | --- | --- | --- |
| Probe position, stimulating E-field orientation |  | Above contact, E-field orientation 0° | Above contact, E-field orientation 45° | Above lead, E-field orientation 0° |
| Distance probe-lead [µm] | 10 | 23 | 19 | 24 |
|  | 500 | 24 | 19 | 24 |

Cassarà, Antonino M., et al. "Safety recommendations for temporal interference stimulation in the brain." *bioRxiv* (2022): 2022-12.

Karimi, Fariba, et al. "Safety of non-invasive brain stimulation in patients with implants: a computational risk assessment." Journal of Neural Engineering (2024).
